# Offline Impedance Measurements for Detection and Mitigation of Dangerous Implant Interactions: An RF Safety Prescreen

**DOI:** 10.1002/mrm.25202

**Published:** 2014-03-12

**Authors:** Christopher W Ellenor, Pascal P Stang, Maryam Etezadi-Amoli, John M Pauly, Greig C Scott

**Affiliations:** 1 Department of Electrical Engineering, Stanford UniversityStanford, California, USA; 2 Procyon Engineering, San JoseCalifornia, USA

**Keywords:** implant safety, pacemakers and MRI, interventional MRI, RF safety, parallel radiofrequency transmission

## Abstract

**Purpose:**

The concept of a “radiofrequency safety prescreen” is investigated, wherein dangerous interactions between radiofrequency fields used in MRI, and conductive implants in patients are detected through impedance changes in the radiofrequency coil.

**Theory:**

The behavior of coupled oscillators is reviewed, and the resulting, observable impedance changes are discussed.

**Methods:**

A birdcage coil is loaded with a static head phantom and a wire phantom with a wire close to its resonant length, the shape, position, and orientation of which can be changed. Interactions are probed with a current sensor and network analyzer.

**Results:**

Impedance spectra show dramatic, unmistakable splitting in cases of strong coupling, and strong correlation is observed between induced current and scattering parameters.

**Conclusions:**

The feasibility of a new, low-power prescreening technique has been demonstrated in a simple phantom experiment, which can unambiguously detect resonant interactions between an implanted wire and an imaging coil. A new technique has also been presented which can detect parallel transmit null modes for the wire. **Magn Reson Med 73:1328–1339, 2015. © 2014 Wiley Periodicals, Inc.**

## INTRODUCTION

Patients with long-wire medical implants, most typically for pacemakers and neural stimulators, are generally prevented from undergoing MRI due to interactions between the implant and the radiofrequency (RF) field. For certain implants and configurations, the RF field can excite currents in the leads causing high local RF current density at the tip, with potential for localized heating 1 and tissue damage 2. The wire under these conditions may be considered as an antenna, the resonant frequency/wavelength and *Q-*factor of which depends on the insulation, the surrounding tissues, and the exposed tip area that acts as a dielectric top-load 3–5. The spatial configuration of the wire will also influence the coupling and resonant properties. This danger is most pronounced when the wire length is close to a resonant length at the imaging frequency 6,7, that can support high antenna currents.

In clinical practice, many circumstances arise: wire implants may be unknown or unreported by the patient [e.g., abandoned leads 8,9] or known wires may have fractured, thereby changing in length and loading condition to create an RF hazard. In neurointerventions, postprocedural retention of microcatheters can occur, many of which contain metallic braiding along some fraction of their length 10–13. Transformer coupling with the main rungs or end-rings can exist when excess lead lengths are coiled under the scalp or elsewhere 14. Without detailed prior knowledge of implant specifics, a major challenge is to help identify and alert potential problem cases even before entry into the magnet. In this article, we propose a prescreen technique to aid in this risk-assessment.

Current techniques to mitigate risk associated with these problems consist simply of protocols that minimize specific absorption rate (SAR). These techniques generally have positive outcomes but compromise achievable image quality and remain controversial 15. A particular failing of low-SAR methods is the wide variation in coupling for different coils, implants, and patient orientations, all of which influence the induced antenna currents on implants and leads. Moreover, improvements in safety are at best linear with decreasing power. Fundamentally, the lack of a reliable method to predict and monitor RF safety/heating of devices in patients leads to both underutilization of MRI where it may be safe, and risk of adverse events in unsafe settings.

Here, we propose a prescreen system scan that can detect dangerous interactions through perturbations of the coil impedance arising from coupling with conductive implants. Such a prescreen could be run on each individual patient before entering the MRI, and similar functionality could also be included within an MRI scanner. This new capability could raise an alert that a more thorough or much more conservative imaging paradigm should be followed, including perhaps extra steps of MRI detection/quantification of tip currents by B_1_ mapping, or artifact detection by reverse polarization if quantitative assessment is necessary.

### Impedance-Based Measurements

Although temperature and RF current are potentially measurable by MRI, neither is a practical physical observable for a rapid prescreening device. If implants are able to support potentially dangerous currents, there is an obvious advantage to screening techniques which use minimal RF power. Network analysis methods can measure the impedance of a coil in a few milliseconds, and with just a few milliwatts of power, which may be considered an ultralow dose. Despite this low power, the impedance of the coil reflects critical electromagnetic properties of its load. Impedance fluctuations are sufficiently sensitive to detect pulse rate and respiratory motion 16,17, and have also been applied to the detection of potentially RF-unsafe devices and conditions. 18–20.

### Parallel Transmit Null Modes

In circularly polarized excitation with an ideal birdcage coil, an instantaneous plane of vanishing electric field 21 rotates spatially at the resonant frequency of the coil. This null *E*-field plane becomes static in the case of a linearly polarized excitation.

The electric field induces dangerous currents in an implanted conductor, and it has been demonstrated that a conductor located in this null plane will experience reduced heating 22. A real coil, however, is unlikely to produce a perfect plane of zero electric field, and the driving phases and amplitudes required to null current generally require some elliptical polarization. In this sense, the birdcage coil may be considered a two-element phased array. As was previously demonstrated with three and four element systems 23–25, and more recently in a birdcage coil 26, the available degrees of freedom of an array may be used to null the electric field in a conductor and suppress current.

This article contains two parts. In the first, we will show that the coupling of a birdcage coil to a potentially dangerous conductive implant produces a measurable effect on the electrical impedance properties of the coil itself, and that these changes are related to induced current on the wire. In the second part, we show that this effect is asymmetric with respect to the phased driving ports of the coil, and that this asymmetry provides information sufficient to prescribe field modes optimized for patient safety.

## THEORY

**Figure 1:**
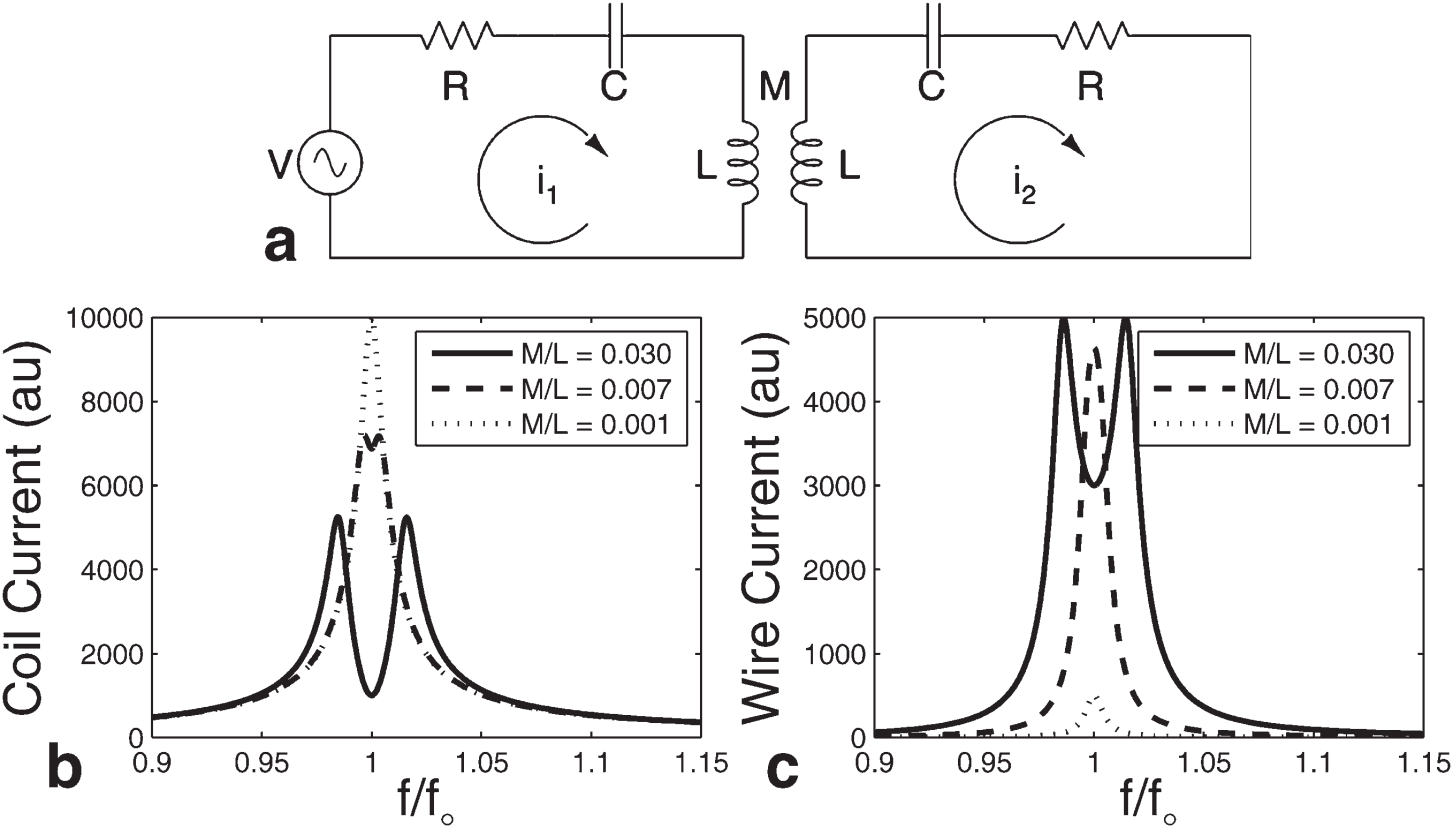
a: A circuit model for the inductive coupling of a resonant wire to a resonant MRI coil. b: As the strength of the coupling increases, the single spectral peak splits into two, and c: current in the wire increases.

In MRI, RF coil loading is typically dominated by inductive and capacitive coupling to the tissue, and is responsible for the well-known broadening of the coil impedance spectrum 27. Embedded wires, however, can exhibit ill-defined resonant properties and couple to the body coil in a fundamentally different way. The combined system of a resonant wire and MRI coil may be thought of very generally as a system of coupled resonators. In coupled systems, whether mechanical, optical, or electrical, the combined oscillation will result in normal modes, each with a slightly different frequency, thus producing a distinct excitation spectrum. To see this, consider the model circuit presented in Figure 1a. The birdcage coil is modeled by the loop on the left, as a voltage source driving a series resonant circuit 28. The wire is represented by the loop on the right, and is driven via an inductive coupling. Two resonant frequencies now exist in the system, the splitting of which can be seen to depend on the strength of the coupling:


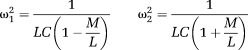
[1]

where *M*, *L*, and *C* are values for the circuit elements shown in Figure 1, representing mutual inductance, inductance, and capacitance, respectively. The figure shows the current resulting in both resonant loops (i.e., the coil and the wire) as a function of excitation frequency, and for different coupling strengths. The single peak splits into two peaks as the coupling is increased, giving an unambiguous sign of resonant coupling. In general, the oscillators may have different resonant frequencies or *Q*-factors (equivalent to *R* in the circuit model), which will tend to reduce the coupled energy and will lead to asymmetrical spectra.

### Coupling Strength and S-Parameters

**Figure 2:**
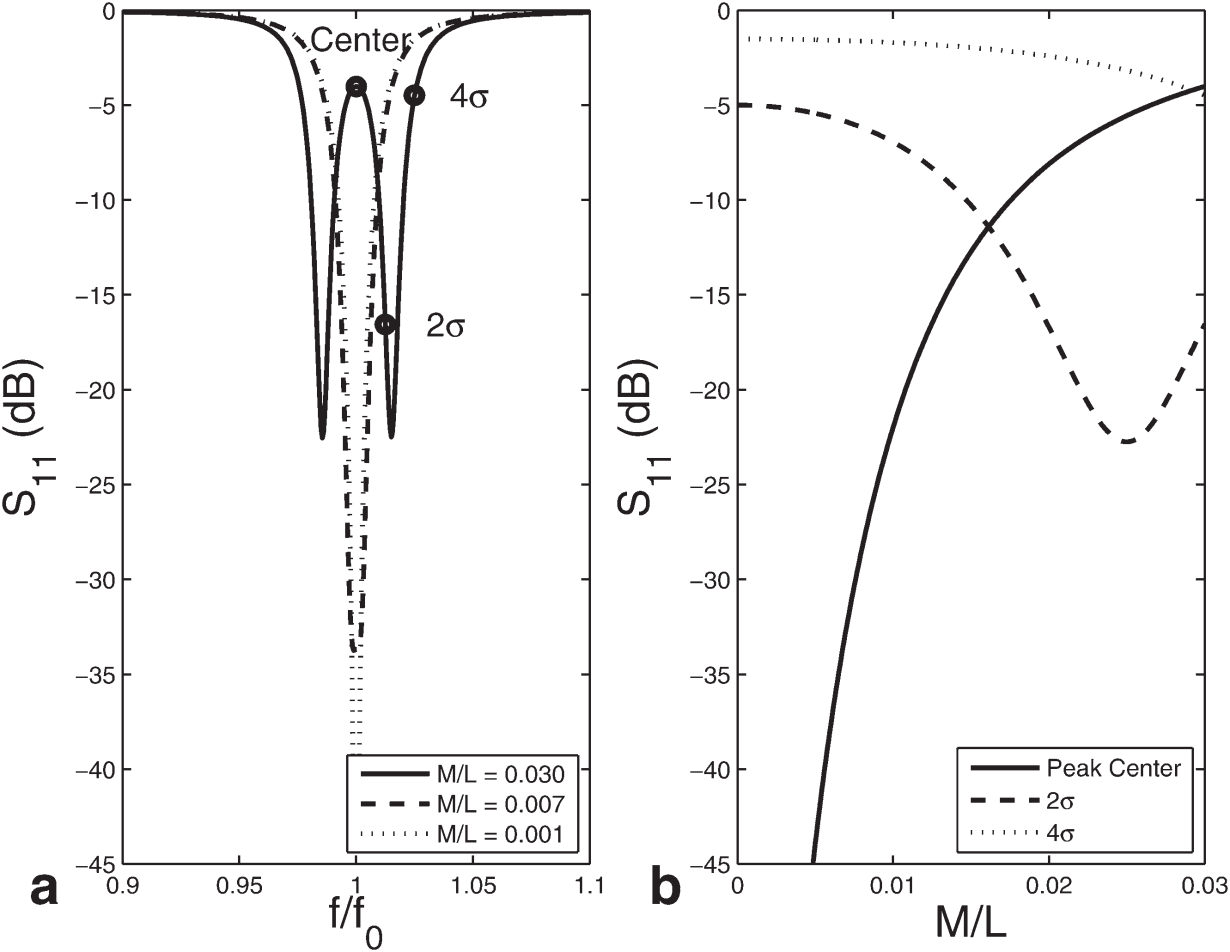
Monitoring reflected power at different frequencies. Panel a shows the *S*-parameter spectrum of the model presented in Figure 1, for three different values of the coupling strength, *M*. Indicated on the *M/L* = 0.030 curve are three points at potential monitoring frequencies, where *σ* represents the HWHM (measured in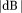) of the unperturbed line shape. Panel b shows the *S*-parameter at these monitoring frequencies as a function of coupling strength. Although nonlinear in coupling strength, the reflected power can be a good indicator of resonant coupling. Care must be taken, however, to choose an appropriate monitoring frequency—the dashed trace shows a case where the derivative changes sign.

If the model presented in Figure 1 is an appropriate model for the wire/coil system, it is expected that the excitation spectrum of the coil will show distinctive features readily identifiable using a low-power scan with a network analyzer. In addition to these “red flag” features, it would also be desirable to have some measure of the coupling strength in order to assess the level of risk. Furthermore, if a specific polarization mode is used to mitigate risk, it would be advantageous to be able to monitor any changes during imaging using a single RF frequency. An approach which monitors the coupling through impedance changes of pickup coils has been presented in 18–20, but here we suggest that the optimal monitoring frequency may not be the imaging frequency. Figure 2 illustrates the change in reflected power for the circuit model given in Figure 1 as the coupling to the resonant load is increased. The behavior is different at different frequencies. Broadly speaking, the result of the interaction is to broaden the spectrum, and to move power away from the center frequency. A probe at the center frequency sees a continuously increasing scattering parameter, and a probe far from the center frequency sees a continuously decreasing scattering parameter (Fig. 2b). Either of these regions is appropriate to monitor the coupling but caution must be used at frequencies in between, as the scattering parameter may increase or decrease with increasing coupling, providing an ambiguous result. In some cases, the spectrum of a loaded imaging coil may be shifted such that imaging is done at such a frequency.

### Position-Dependent Coupling

The birdcage coil with its two drive ports may be treated as a two-channel phased array whose resonators and their couplings to the wire implant may be considered separately. The electric field distribution of the birdcage coil at its first-order resonance is well-known 21, and we consider here a two-dimensional model representing an axial cross section. For a linearly polarized mode, the electric field is oriented along the coil's axis (*z*), and the spatial dependence of its magnitude may be written as


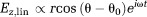
[2]

where *r* is the radial position, θ is the angular position within the coil volume,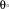 denotes the location of maximal rung current, and *ω* is the oscillation frequency. From this formulation, it is clear that in the plane where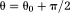, the electric field is zero. Furthermore, by adding a second drive port at a different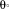 (usually 90° separated), a linear combination of fields can be created such that the plane of zero electric field can be arbitrarily oriented. Because the current induction on a conductive implant will be proportional to the electric field it experiences, the angular location,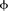, of a long, *z*-oriented conductor in the coil is recoverable by measuring the ratio of the inductive coupling strengths to each channel.


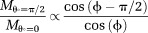
[3]

where *M* is the magnitude of the inductive coupling. To obtain this ratio of *M* values, the deviations of the two *S*-parameters are measured at frequencies where each varies approximately linearly with the inductive coupling (although this is an idealization, see Fig. 2). The recoverable angle is thus


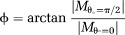
[4]

yielding a fourfold symmetry, and defining two orthogonal planes within the coil. To reduce the symmetry, we observe that each drive port of the birdcage drives a mode in which the electric field has opposite phase across the so-called *E* = 0 plane. An implant-generated coupling between the two drive ports (i.e., the *S*_12_ parameter) can, therefore, be used to assign two quadrants within the coil, because its phase will be determined by the parity of the phases of the driving fields. One of the previously determined planes will lie in these quadrants, the other will not.

In general, an implant will not be straight and oriented along the long axis of a birdcage coil, and moreover, it is not particularly important to locate an implant spatially. We offer this as a particularly intuitive demonstration of how *S*-parameter measurements can be used. This concept will apply more generally to phased arrays, where using *S*-parameter derived knowledge of the coupling strengths, one drive channel can be configured to counteract the effect of another.

## METHODS

### Coil

**Figure 3:**
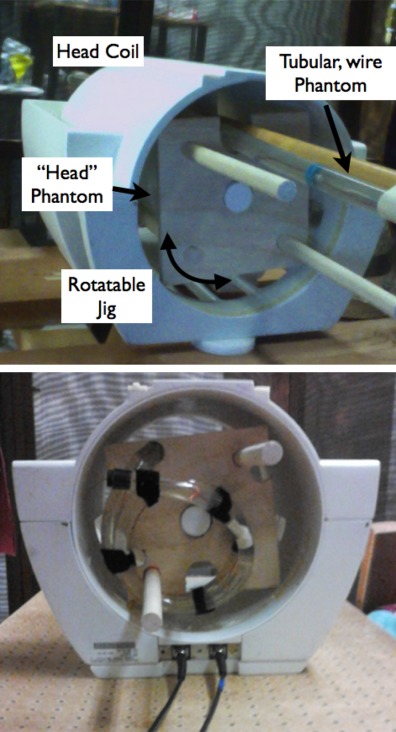
Phantom and coil setup for the experiments. A tubular wire phantom is mounted on a cylindrical head phantom using a rotatable jig. The upper photo shows the straight wire phantom (Fig. 5, Configuration e) and the lower photo shows the wire phantom with two off-center loops at the end (Fig. 5, Configuration f).

For our study, we use a modified birdcage head coil, pictured in Figure 3, which we have used previously to study reverse polarization imaging 29. The two ports of the birdcage coil can be excited independently to generate arbitrary field modes—linear, circular, or elliptical. We use a Medusa console 30 to simultaneously excite the two ports with arbitrary phase and amplitude. For this experiment, this independence also allows the impedance of each drive port of the coil to be measured individually. The coil includes a matching network, and is thereby tuned to have a 50-Ω impedance at 63.9 MHz, although deviations are observed in the course of the experiment due to different loading and shielding conditions. The modification of the birdcage coil has also produced some distortion in the unperturbed *S*-parameter spectrum, which will be discussed further in the Results section.

### Phantom

To simulate loading of the head coil by tissue, a cylindrical phantom was placed along the *z*-axis of the coil. The phantom had a volume of 3.8 L, a diameter of 156 mm, and contained a 0.5 mmol MgCl_2_ solution, as well as 2.5 g/L NaCl to produce a conductivity of 0.5 S/m. A flexible tubular phantom of length 1.5 m and diameter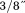 was filled with the same solution and mounted 1 cm from the edge of the head phantom. Inside the tube, an AWG16 copper magnet wire ran along its center, extending 10 cm from either end of the phantom. The length of wire extending beyond the phantom was cut so that that the wire would be of a near resonant length in the 64-MHz imaging field. This was determined empirically by maximizing the spectral distortion for the straight, axially oriented wire phantom. This exposed span of wire was also used to mount our custom current sensor, described below. The system was constructed to be cylindrically symmetric about the coil axis, except for the wire phantom, and to be rotatable about this axis. The flexible tubular phantom was kept physically separate from the cylindrical phantom to be easily configurable into different shapes.

### Current Detection

**Figure 4:**
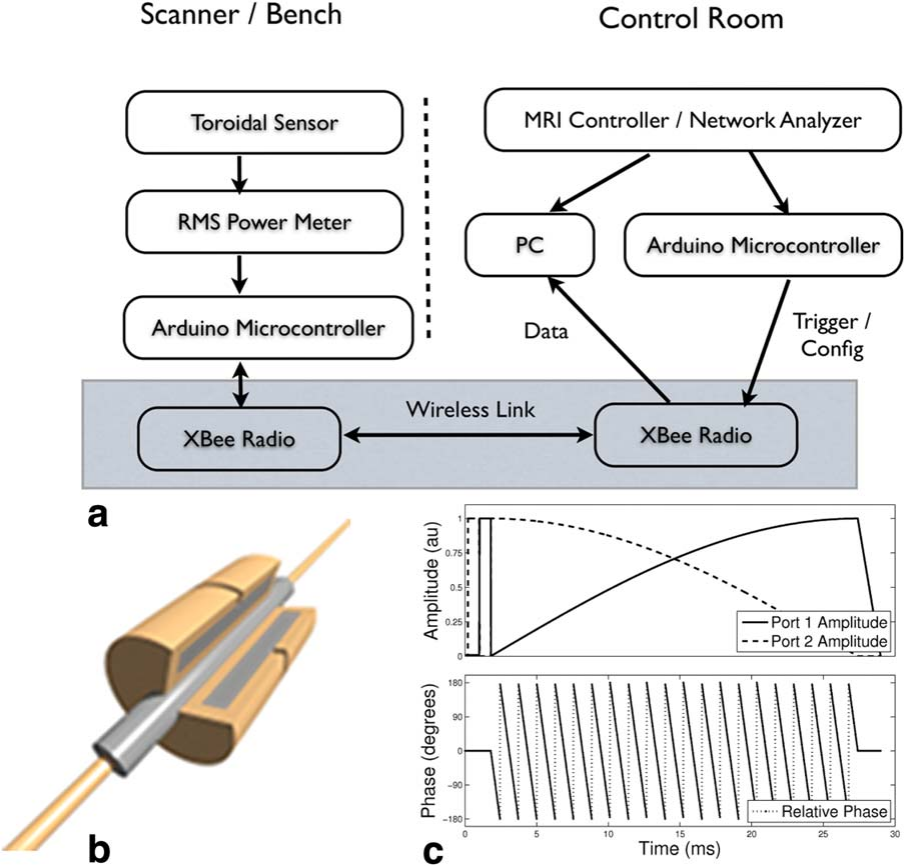
Experimental setup for measuring wire current. a: The system diagram for wireless transmission of current data. Signal from the current sensor is converted with an RF RMS power sensor, digitized and transmitted using an XBee 2.4 GHz radio transceiver. b: A Cut away of current sensor used to measure current in a wire. The sensor is a volume-rotated pickup loop which couples flux due to current in the wire. Teflon tubing is used to insulate the sensor from the wire, as well as to fill the gap in the loop. The sensor used is approximately 1 cm in diameter and 5 cm in length. c: Waveforms used to drive the two ports of the birdcage coil while in the scanner. The two ports are initially pulsed independently, allowing for the acquisition of the two-port scattering parameters. The relative phases and amplitudes of the two channels are then swept to sample the response to composite signals over a broad region of parameter space.

To demonstrate that a characteristic distortion of the impedance spectrum is a clear indication of coupling to a wire, and therefore, induced current, it is important to have an independent measure of induced current. For this purpose, we use physical current sensors which measure the current in a wire. The toroidal sensors (Fig. 4b) are in the form of a pickup loop, volume-rotated about the wire, and have been described in 31,32. The sensors act as transformers whose coupling to the wire can be varied by toroid length and inner/outer diameter. The sensors are slid over the portion of the wire protruding from the tubular phantom, measuring the current approximately 5 cm from the end of the wire. As the current along the wire is nonuniform, and the relationship between current in the free-space and submersed portions can be difficult to predict 33, we understand the reading only as a proportional measure of the current in the wire current mode.

This work makes use of two versions of these toroidal sensor systems. The first version outputs the sensing voltage via coaxial cable, and is used for network analyzer measurements where the phase of the current is of interest. The second version of the system, illustrated in Figure 4a, eliminates the coaxial cable and transmits current data wirelessly to the control room. An RF power sensor integrated circuit (Analog Devices, AD8361) is placed at the base of the toroid to measure the current magnitude. The power output is then digitized by an analog-to-digital converter (ADC) on board an Arduino Pro 328 (SparkFun Electronics, Boulder, CO) microcontroller, and transmitted wirelessly to the console using an XBee (Digi International) radio transceiver. The measurement of current magnitude is, therefore, completely wireless, avoiding the confounding induction of common-mode currents on long coaxial cables. However, unlike the optical fiber approach of 31,32 which allows full coherent detection, the AD8361 loses all phase information during the power measurement. While this is important in some measurements, such as identifying normal modes of the composite system, it can be neglected when only the magnitude of the current is required. Although the *S*-parameters alone should suffice, the relative phase of the coupling to the wire for each drive port may be directly visualized through the use of a novel pulse shown in Figure 4c. Power to one channel is increased, whereas power to the second channel is decreased. Simultaneously, the relative phase of the drive channels is rapidly varied, in effect sampling the entire space of phase/amplitude combinations in a brief pulse. This waveform is pulsed at approximately 100 mW.

### First Experiment––Offline Prescreen

The goal of the first experiment is to demonstrate that a spectral scan of coil *S*-parameters can clearly reveal the existence of a resonant, conducting implant, without the need for a full scan, or even the need to be near a scanner. This experiment was performed with a network analyzer on a laboratory bench, and is pictured in Figure 3.

The network analyzer (Agilent E5071C, Santa Clara) measured the two-port *S*-parameters of the drive ports of the birdcage coil for different azimuthal positions of the wire phantoms, approximately 4 cm from the inner diameter of the coil. Data were collected in the range of 55–75 MHz, with the wire at evenly spaced locations around the coil diameter. The power output of the network analyzer for these experiments is typically set to 1 mW, and in some weak-coupling configurations to 10 mW, both well below the threshold for dangerous interactions. All presented data have been normalized with respect to drive power.

### Nonstraight Wires

**Figure 5:**
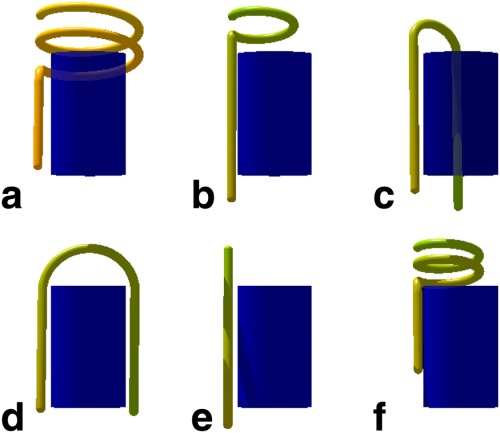
Wire shapes studied (a) large double loop, (b) single loop, (c) 90° “U,” (d) 180° “U,” (e) straight, and (f) small double loop. The blue rectangle represents the cylindrical head phantom, and the yellow cylinder represents the tubular phantom containing a wire and saline solution.

Although amenable to understanding with simple physical models, the case of a long, straight wire is unlikely to occur in real patients. For this reason, we have conducted experiments on irregularly shaped wire phantoms. Generally, we expect that the current generated in the wire will be the result of the one-dimensional vector sum of the stimuli from each drive port, and should not be distinguishable from a straight wire in our experiment. Figure 5 illustrates six different configurations that tested the ability of impedance measurements to predict dangerous currents. Of particular interest is the case where a long, straight wire is terminated in a coil, as is typically done to take up excess lead length of a neurostimulator, and may act as an additional voltage source by coupling magnetic flux generated by end-ring currents in the coil.

### Second Experiment––Scanner Validation and Current Suppression

The second experiment aims to demonstrate the transferability of the offline prescreen to the MRI environment, and also to show how impedance information can be used to identify modes of the driving field that can suppress induced currents. The proposed measurement will be valuable not only as a prescreen, but also as a monitoring tool, as coupling strengths could change during a scan due to many factors. Examples include motion of the patient or interventional instrumentation, or the drifting of electrical properties due to temperature change. A GE Signa 1.5T (Milwaukee, WI) scanner was used with the same phantom described in the previous experiment. In this case, the coil impedance was detected at a single frequency, using the Medusa console 30 and directional couplers (MiniCircuits ZFBDC20-62HP+) in the RF chain as a network analyzer. In this way, the *S*-parameter measurement can be integrated directly into the scan sequence. A pulse as shown in Figure 4c is delivered to the coil. The power is set as low as possible while still producing a measurable current signal. The response of the coil to this sweep is predictable, in principle, by the linear combination of the *S*-parameters. The current response, however, must be measured with a full sweep as our root mean square sensor does not preserve phase information, and complex *S*-Parameters cannot be recovered.

Peak powers for the data presented in the second experiment are approximately 100 mW, several orders of magnitude below a typical MRI scan.

## RESULTS

### First Experiment––Coil Spectra

**Figure 6:**
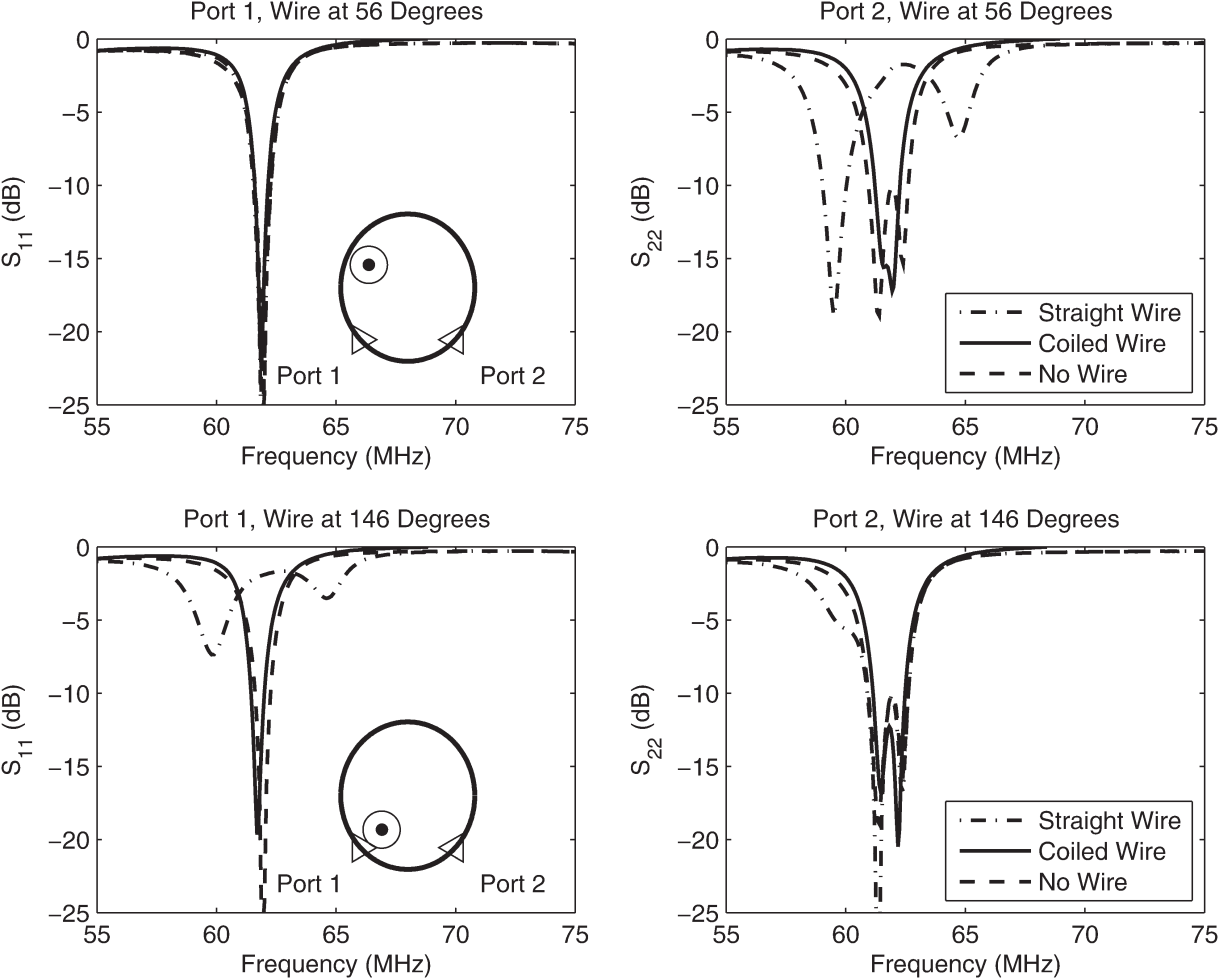
*S*-parameter spectra of a birdcage coil loaded with a head phantom and a tubular wire phantom in two configurations (Fig. 5e,f), at two different locations. The third curve shows the coil spectrum with only the head phantom. The spectra show obvious distortion when the coil is a loaded with a resonant load, and the increased distortion of the straight-wire phantom is shown to correspond to greater (≈4×) induced current (Fig. 7). The distortion is asymmetric between drive ports, allowing for the identification of minimally interacting polarization modes. Triangles on the coil schematics indicate the positions of the drive ports, where *E* = 0 planes are expected for ideal coils.

Figure 6 shows the *S*-parameter magnitude spectra for each port of a birdcage head coil with the wire phantom in two different configurations––straight and coiled (Fig. 5e,f) in two different angular positions. The spectrum of the head phantom with no wire is also shown. For each wire position shown, one port clearly shows distortion while the other port shows a spectrum nearly identical to that due to the reference phantom. The spectral splitting described in the Theory section is clearly visible for the straight-wire phantom, whereas the coiled-wire phantom shows only minor distortion. It will be shown below that this is a result of reduced coupling of the wire, and corresponds to less induced current. For each of the angular positions shown, the wire has been located approximately in the *E* = 0 plane of one port, which is expected to be near the drive point of the opposite port. We note that the spectrum of coil port 2 contains a double peak even without a resonant load present. This appears to be due to its modification to be dual-drive. We note by comparison to the other port that the broad features of the wire interaction appear unaffected, but detailed features show anomalous behavior near the peak center and should not be interpreted quantitatively.

A study of other wire configurations, depicted in Figure 5, shows spectral distortion qualitatively similar to those presented in Figure 6, but of varying severity. To generalize this effect, we introduce a “distortion parameter,” defined as


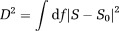
[5]

where *S* is the *S*-parameter spectrum of the system under study, *S*_0_ is the spectrum of the reference phantom, and *f* is the frequency parameter over which the spectrum is considered, which will be chosen to span the significant portion of the reference phantom spectrum. For a given patient, spectral distortion may be produced by both resonant wire coupling and the usual dielectric loading, and by comparison with a completely empty coil, we find the magnitude of these effects to be on the same order (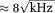, integrated from 60.5 to 63.5 MHz).

To understand the expected variation of the distortion parameter from patient-to-patient, based solely on dielectric loading, eight volunteers placed their heads in the coil, and the distortion parameter was measured versus an empty coil. The measured distortion parameters were about the same as for the head phantom, with a standard deviation of around 5% of the mean value. This variation is thus small compared to the effect to be measured. Furthermore, dielectric loading never shows the distinctive features present in a resonantly coupled spectrum—the most dangerous case. We expect that the distortion parameter will increase monotonically with increasing coupling, based on the expected spectral distortion outlined in Theory section.

**Figure 7:**
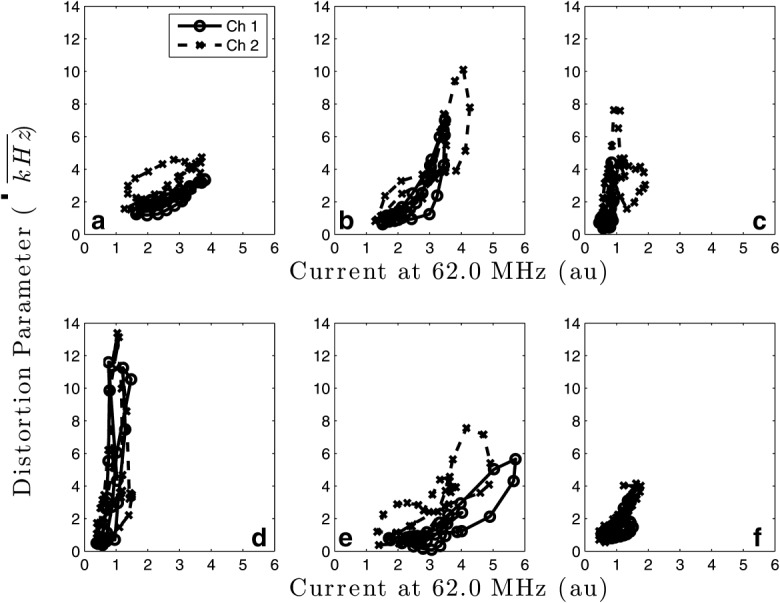
Relationship of wire current to spectral distortion. Study of different wire phantom configurations at various azimuthal angles shows a strong correlation between spectral distortion (Eq. [5]) and current induced in the wire. Panels correspond to wire configurations shown in Figure 5, and spectra have been integrated from 60.5 to 63.5 MHz, reflecting the shifted resonant peak of the bench experiment.

Figure 7 shows this distortion parameter plotted versus induced wire current for various wire configurations. The distortion parameter varies nearly linearly with induced current, although slope and scatter vary considerably. We conclude that the distortion parameter may be useful to spot changes in coupling during a scan, but the variation in slope among configurations renders difficult any absolute estimate of coupling.

### Phantom Location

As described in Theory section, the strength of the coupling between the birdcage resonator and a straight wire will depend on the spatial position of the wire. Furthermore, the strength of the interaction can be estimated through measurement of the coil *S*-parameters, and it is, therefore, possible to determine the angular wire position from *S*-parameter measurements. To demonstrate this, the locations of a straight wire and a coiled wire load have been reconstructed. *S*-parameter magnitudes were collected, the reference values subtracted and Eq. [4] is applied to recover the position of the wire.

**Figure 8:**
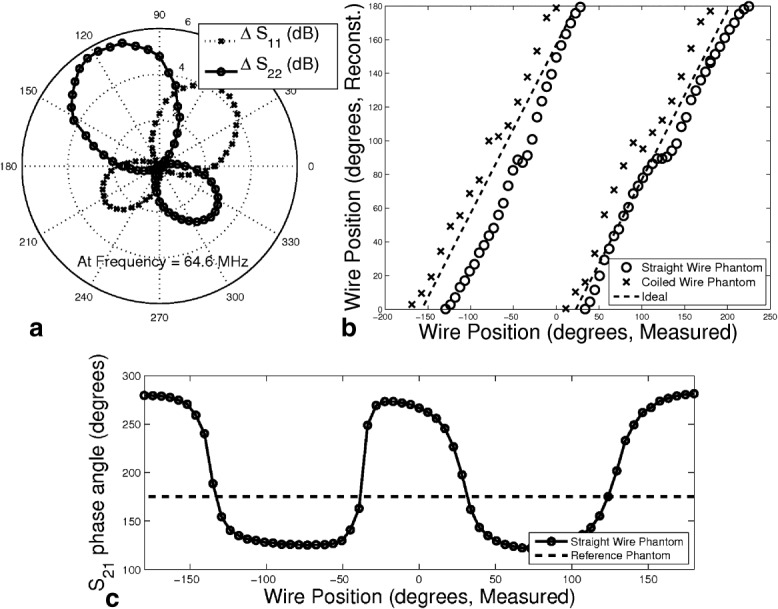
*S*-parameter measurements and wire position reconstruction. Panel (a) shows *S*_11_ and *S*_22_ for the straight wire phantom (Fig. 5e) with values for the reference phantom subtracted. Panel (b) shows reconstructed wire position for the wire phantom in configurations e and f plotted against the actual position; the dashed line shows the ideal relationship. Panel (c) shows the angle of the *S*_21_ parameter for configuration e, where a value of 180° is expected for a nonresonant load.

Figure 8 shows the impedance-estimated angular location of the two wire phantoms in the phantom rotation experiment, as well as the measured values. An offset has been subtracted from the reconstructed values due to the uncertainty of the effective drive points of the coil. Using just the impedance measurements, the location of the plane containing the wire has been determined. An important difference was observed, however, between the straight and coiled wires. In the case of the straight wire, the fourfold symmetry of the distortion ratios can be easily reduced to a twofold symmetry by reference to the *S*_21_ parameter, due to a clear model for scattering between channels. In the case of the coiled wire, the coupling is more complicated, and may include significant interactions with the end ring. The bottom panel in Figure 8 shows the phase of the coupling between channels for the straight wire, which behaves as expected and allows a point-by-point reconstruction of wire position. The coupling for the coiled wire is more complex, and the position reconstruction has been by reference to the entire dataset.

Notably, the location of the wire in spatial coordinates is not necessarily significant in detecting and suppressing dangerous currents, but rather it is the comparative coupling to the drive ports. Due to the symmetry of the birdcage coil, the relative coupling has a spatial interpretation, and we include this as a vivid demonstration of the potential of this method.

### Second Experiment––In Scanner

**Figure 9:**
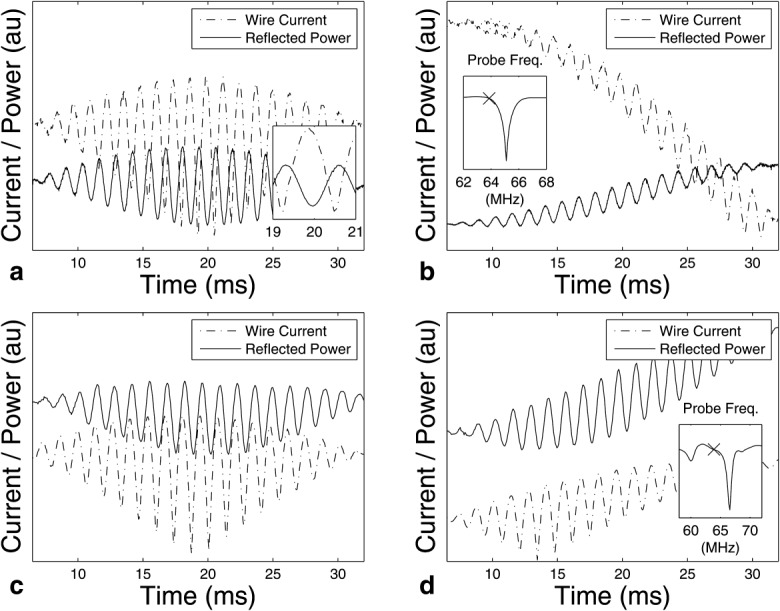
Reflected power as a proxy for induced current. The results of the pulse are shown in Figure 4c, where intensity is swept from one channel to the other, with a faster phase modulation between ports. Panels a and b show the results for a coiled wire in two positions, one coupled approximately equally to the drive ports (a), and the other coupled more strongly to one (b). The reflected power can be used to locate the amplitude/phase combinations needed to null induced current. The inset in panel a shows a closer view of the current and reflected power, where slight phase discrepancy can be seen. The inset in panel b shows the *S*-parameter spectrum of the coil loaded with the reference phantom (port 1, see Fig. 6), with a mark indicating the frequency at which reflected power is monitored. Panels (c) and (d) show similar results for a straight wire, although the sign of the correlation is reversed. The inset in panel d shows *S*-parameter spectrum in the case of maximal coupling to the long wire, with the probe frequency indicated.

Figure 9a shows an example of data from the rotated field experiment, where the wire phantom with the tight double-loop (*F*) was placed at a single location and the parameter space of imaging fields was scanned. The current measured oscillates rapidly as the relative phasing between the channels is swept, the local minima occurring where the two channels excite current of opposite phase. A slowly varying envelop is also apparent, allowing for the determination of the correct amplitude ratio between the channels. This visualization pulse confirms that the drive fields can be manipulated to suppress current. The second curve measures the total reflected power from the coil, where the individual channels have been normalized due to differences in their unperturbed *S*-parameter spectra.

The reflected field from a given port is the sum of the reflected field from its drive point, plus that coupled from the other port primarily via the wire. The reflected power is the coherent sum squared. For a two-port excitation which nulls current, reflected power at a port is at a maximum when these two fields are in phase and at a minimum when these fields are of opposite phase. It is the relative phase of the coil and wire currents then, which determines the sign of the correlation, and this relative phase depends on the drive frequency relative to the resonance curve, as well as the sign of the coupling to the birdcage coil (determinable from *S*_21_). If the coupling between wire and coil has a different phase shift for the different ports, a slight phase shift will occur in the scattered power, as pictured in the inset of Figure 9a.

Figure 9b shows a second example where the wire is more strongly coupled to one channel than the other. Here, the null mode will be driven mostly by the uncoupled channel, and the relative phase of the channels produces much smaller modulations.

Figure 9c,d shows similar results for the case of a straight wire, although the sign of the correlation has been reversed. The monitoring frequency sits between the two peaks of the split spectrum and where the wire current is expected to lag coil current by 90°, and the scattering parameter increases as coupling increases.

This demonstrates the crucial result: that by simply monitoring the power transmitted through the RF chain, and using no additional sensors, it is possible to identify polarization modes which suppress (or enhance) current induced on implanted wires. Moreover, because the coupling scan is extremely short and extremely low power, it can be performed repeatedly during an imaging scan—even every few seconds—without significantly prolonging the procedure. Continual monitoring can be used to ensure that patient motion or drift of electrical properties have not changed the coupling between wire and coil.

**Figure 10:**
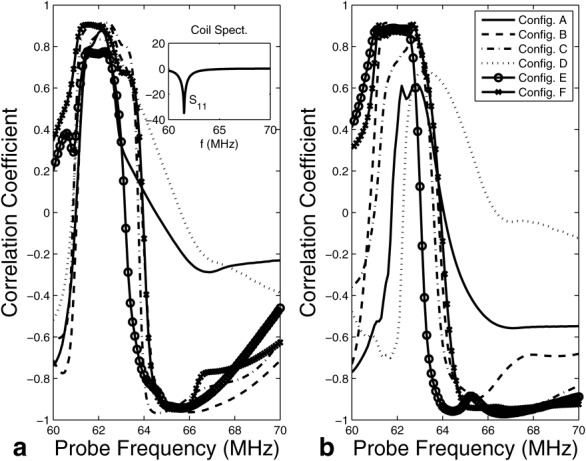
Correlation coefficients (Pearson's *R*) between wire current and change in *S*-parameter magnitude (vs. reference phantom) for the two drive ports (panels a and b), in the six configurations shown in Figure 5, measured offline with a network analyzer. Each configuration shows regions of robust correlation, although the location of the region varies. The inset shows the *S*-parameter spectrum, and the correlation follows that expected from Figure 2 with the correlation being strong and positive at the peak, strong and negative on the wings and ambiguous in between.

In general, all configurations showed strong correlations between induced current and *S*-parameter magnitude at some frequencies, although that frequency range varies. These correlation results are summarized for the bench top experiments in Figure 10, where the correlation coefficient is shown as a function of monitoring frequency. The Pearson *R* coefficient, which is calculated between wire current and the change in *S*-parameter magnitude (vs. the reference), has been plotted to highlight the change in both the strength and sign of the correlation. An *R* with large absolute value implies a linear relationship (which need not exist––see Fig. 2b), and in these regions we expect *S*-parameter monitoring to be indicative of current coupling. An *R* with small absolute value indicates a situation where there is little correlation between current and *S*-parameter, and these regions cannot be used for monitoring.

## DISCUSSION

It has long been recognized that implant recipients can incur the added risk of RF heating local to the electrode tips during MRI. Any prescreening device that could aid in gauging risk before scanning would be an important development. Yet, we have a catch-22: neither measures of induced RF lead currents that drive heating nor the resulting temperature are accessible without invasive probes or robust MRI RF field and thermal mapping methods. We are obliged to identify accessible physical observables that could help triage these interactions before MRI scanning.

The technique demonstrated in this article directly measures interactions with wire implants, without need for prior knowledge of the configuration or length of the implant. This capability could even be performed offline using an apparatus distinct from the MRI scanner. A safety prescreen system external to or within an MRI scanner could be of great value in screening patients with known or unknown potentially dangerous implants. Information on the strength and nature of the coupling can be obtained in an extremely safe manner and requires only a few milliseconds and a few milliwatts of RF power.

A similar detection scheme has been demonstrated elsewhere using pickup coils to measure load perturbations both with and without a reference scan 18–20. It should be considered complementary to the work presented here. Our approach differs significantly in that we consider coil properties at more than just one frequency. As we have shown, the study of coil impedance over a spectrum can give important information pertaining to both the strength and the nature of the coupling that may not be obvious at a single frequency. Moreover, we have shown that to perform continuous monitoring the imaging frequency is in some cases not the best choice for tracking coupling strength.

By treating the birdcage coil as a phased array, this work adds the ability to prescribe polarization states for subsequent scans which minimize induced current. It has been shown previously 22 that dangerous heating in implants can be significantly reduced through the use of appropriately polarized RF fields, and more recent work has demonstrated an image-based technique to identify the optimal polarization 26. The work presented here demonstrates a faster, lower-power method to detect the optimal field mode for imaging. The reduction in coupling achieved by this optimal mode can be estimated, and the advantage weighed against the penalty incurred in overall SAR. The application of the null mode detection sweep in larger parallel transmit arrays could be done between any two linear combinations of elements, and would allow reduced perturbation to the transmit field as more degrees of freedom are available to both null the current and create an optimized excitation 23,24,34.

The prescreen can identify dangerous interactions and strategies to mitigate them, but the coupling of a wire implant to a MR field also depends on the position of the implant in the scanner. If a parallel transmit null imaging mode is prescribed and used, the same prescreening scan should be performed regularly in the scanner to monitor any change in coupling due to patient motion or landmark changes, and respond programmatically with the RF drivers. The ability to do “live” measurements, where the polarization can be dynamically determined and adjusted could also be invaluable in interventional procedures.

A scan to be performed with a nulling polarization mode could proceed as follows. The coil *S*-parameter spectrum is acquired (or retrieved) for the case of loading with a suitable reference phantom, with size, position, and electrical properties similar to the subject. This is the baseline for study of the actual patient, and by referring to the acquired spectra and assuming behavior as described in the model above, suitable monitoring frequencies are chosen for the drive ports, where *S*-parameter values are expected to vary monotonically (as in Fig. 2a). The patient then enters the scanner, or the offline mockup. A parameter space scan is performed (i.e., as in Fig. 4c), and the measured coil properties are compared to those of the reference phantom. Depending on the sign of the correlation at the frequency chosen, the configuration producing maximum or minimum power reflected from the drive ports is the nulling scanning mode. This potentially safer configuration could be determined during the offline prescreen and transferred to the scan console, or could be measured and monitored by the scanner itself.

## CONCLUSIONS

In this work, we have identified the distortion of RF coil *S*-parameter spectra as a suitable offline prescreening metric. These spectra are practically measurable on all people at mW power levels, are noninvasive and can be performed independently of MRI scanning. Our experiments with a dual-port-drive birdcage head coil demonstrate that wire coupling creates detectable perturbations in *S*-parameter spectra collected in a 20-MHz bandwidth. These interactions correlate with induced RF current as detected by RF current sensors using drive levels of 10 mW or less and a variety of curvilinear wire structures. A distortion parameter metric was also proposed using the integrated absolute *S*_11_ and *S*_22_ deviation over bandwidth. It was shown in all configurations tested that this metric varied nearly linearly with induced wire current over some range, and could be of use for monitoring changes in coupling during a scan. Furthermore, we have shown that by probing coil *S*-parameters for different transmit configurations, it became possible to identify polarization modes which suppress (or enhance) current induced on wires using timescales under 50 ms and drive powers of 100 mW.

Many pathways for development and validation remain to be explored. Enhancements such as pattern matching of the *S* or *Z* spectra are possible, and nonimaging structures designed specifically to detect buried resonant devices should be investigated. Potentially, the MR transmit chain could incorporate a broadband network analysis functionality before and during scanning. Finally, there could be significant utility in outfitting MR facilities with a separate, dedicated prescreening system comprising a mock imaging coil network analysis system, outside the MR scanning suite.

We conclude that RF coil impedance-based perturbations could be a viable metric to prescreen patients for potentially dangerous RF interactions in wire-like implants, or to aid rapid identification of safer transmit polarization modes. This new capability could alert the clinician that a more thorough or much more conservative imaging paradigm should be followed. If quantitative assessment is deemed necessary, additional MRI scans could then be acquired that detect artifacts by low power forward or reverse polarization, or quantify tip currents by B_1_ mapping.
